# *Cytophaga hutchinsonii* SprA and SprT Are Essential Components of the Type IX Secretion System Required for Ca^2+^ Acquisition, Cellulose Degradation, and Cell Motility

**DOI:** 10.3389/fmicb.2021.628555

**Published:** 2021-02-12

**Authors:** Lijuan Gao, Yahong Tan, Weican Zhang, Qingsheng Qi, Xuemei Lu

**Affiliations:** State Key Laboratory of Microbial Technology, Shandong University, Qingdao, China

**Keywords:** ion acquisition, T9SS, Ca^2+^ acquisition, cellulose degradation, cell motility, protein secretion

## Abstract

The type IX secretion system (T9SS) is a novel protein secretion system, which is found in and confined to the phylum *Bacteroidetes*. T9SS is involved in the secretion of virulence factors, cell surface adhesins, and complex biopolymer degrading enzymes to the cell surface or extracellular medium. *Cytophaga hutchinsonii* is a widely distributed bacterium, which is able to efficiently digest cellulose and rapidly glide along the solid surfaces. *C. hutchinsonii* has a full set of orthologs of T9SS components. However, the functions of most homologous proteins have not been verified. In *C*. *hutchinsonii*, CHU_0029 and CHU_2709 are similar in sequence to *Flavobacterium johnsoniae* T9SS components SprA and SprT, respectively. In this study, the single deletion mutants of *chu_0029* (*sprA*) and *chu_2709* (*sprT*) were obtained using a complex medium with the addition of Ca^2+^ and Mg^2+^. Single deletion of *sprA* or *sprT* resulted in defects in cellulose utilization and gliding motility. Moreover, the Δ*sprA* and Δ*sprT* mutants showed growth defects in Ca^2+^- and Mg^2+^-deficient media. The results of ICP-MS test showed that both the whole cell and intracellular concentrations of Ca^2+^ were dramatically reduced in the Δ*sprA* and Δ*sprT* mutants, indicating that SprA and SprT are both important for the assimilation of trace amount of Ca^2+^. While the assimilation of Mg^2+^ was not obviously influenced in the Δ*sprA* and Δ*sprT* mutants. Through proteomics analysis of the cell surface proteins of the wild type and mutants, we found that the Δ*sprA* and Δ*sprT* mutants were defective in secretion of the majority of T9SS substrates. Together, these results indicate that SprA and SprT are both essential components of *C*. *hutchinsonii* T9SS, which is required for protein secretion, Ca^2+^ acquisition, cellulose degradation, and gliding motility in *C. hutchinsonii.* Our study shed more light on the functions of SprA and SprT in T9SS, and further proved the link between the T9SS and Ca^2+^ uptake system.

## Introduction

*Cytophaga hutchinsonii* is a widely distributed bacterium belonging to the phylum *Bacteroidetes*, which is able to efficiently digest cellulose and rapidly glide along the solid surfaces (Stanier, 1942; Xie et al., 2007). The cellulose degradation mechanism of *C. hutchinsonii* is distinct from the free soluble cellulolytic enzymes and multiprotein cellulosome (Lynd et al., 2002; Bayer et al., 2004; Wilson, 2008, 2011). Direct contact with cellulose is essential for cellulose degradation by *C. hutchinsonii*, and most of the cellulase activity seems to be cell associated (Chang and Thayer, 1977). No genes encoding cellobiohydrolases or proteins with typical carbohydrate binding module (CBM) were identified in the genome of *C. hutchinsonii* (Boraston et al., 2004; Xie et al., 2007). The cellulose degradation mechanism of *C. hutchinsonii* is still mysterious. It is speculated that the outer membrane proteins, which could contact with cellulose or its hydrolysate, may play important roles in the utilization of cellulose by *C. hutchinsonii* (Zhu and McBride, 2017; Wang et al., 2019). Specifically, CHU_1276, CHU_1277, and CHU_3220, which are outer membrane proteins, are essential for the utilization of crystalline cellulose (Ji et al., 2014; Zhou et al., 2016; Wang S. et al., 2017).

Gram-negative bacteria have evolved several protein secretion systems to translocate substrates across the outer membrane (Rego et al., 2010). The type IX secretion system (T9SS) is a novel protein secretion system, which is widespread in and confined to the phylum *Bacteroidetes* (McBride and Zhu, 2013). The T9SS is essential for the formation of cell surface coatings, which play vital roles in pathogenicity, utilization of polysaccharides including cellulose, and cell motility (Veith et al., 2017). Substrates of the T9SS contain N-terminal signal peptides and conserved C-terminal domains (CTDs). These proteins rely on the Sec transport system to cross the cytoplasmic membrane, then are directed to the T9SS by CTD. The T9SS has been extensively studied in *Porphyromonas gingivalis*, a human oral pathogen, and *Flavobacterium johnsoniae*, a widespread gliding bacterium (Lasica et al., 2017). At least 19 proteins were identified to be the components of T9SS. Although general functions of most T9SS components have been reported (Gorasia et al., 2020), how this multiprotein machinery works is still unclear. Previous work reported that PorK, PorL, PorM, and PorN, which are the core components of T9SS, are essential for the secretion of gingipains in *P. gingivalis* (Sato et al., 2010). These four proteins oligomerize and interact through a dense network of contacts forming a large secretion apparatus that spans the cell envelope (Vincent et al., 2017). PorT (ortholog of SprT), which is indispensable for the maturation and transportation of gingipains in *P. gingivalis*, was found as the first component of T9SS in 2005 (Sato et al., 2005). Then, Sov (ortholog of SprA) was found to be essential for the secretion of gingipains in *P. gingivalis* in 2007 (Saiki and Konishi, 2007). Furthermore, SprA is required for the secretion of SprB, RemA, and ChiA (enzyme required for chitin digestion) in *F. johnsoniae* (Shrivastava et al., 2013). In 2018, the structure of SprA was solved by cryo-electron microscopy, revealing that SprA is the outer membrane translocon of T9SS in *F. johnsoniae* (Lauber et al., 2018).

Homologous genes encoding components of the protein secretion systems (T1SS, T3SS, T4SS, T5SS, T6SS, T7SS, the ENP pathway involved in biogenesis of curli, the chaperone-usher pathway involved in pilus assembly) found in other bacteria are lacking in the genome of *C. hutchinsonii*. Four genes encoding the major components (T2S-D, -E, -F, and -G) of the T2SS were identified in the genome of *C. hutchinsonii* (Xie et al., 2007; McBride and Zhu, 2013). Individual disruption of the three T2SS genes (*T2S-D*, *-F*, and *-G*) results in a significantly retarded growth on cellobiose, regenerated amorphous cellulose, and Avicel cellulose (Wang X. et al., 2017). *C. hutchinsonii* has a full set of orthologs of T9SS components (Sato et al., 2010; McBride and Zhu, 2013; Wang et al., 2014). Furthermore, genome scanning of *C. hutchinsonii* found at least 147 proteins with conserved CTDs (Veith et al., 2013; Zhu and McBride, 2014). Previous works showed that CHU_3237 (PorU) and CHU_0170 (SprP) are components of *C. hutchinsonii* T9SS. Single deletion of *chu_3237* (*porU*) or *chu_0170* (*sprP*) caused defects in cellulose degradation and cell motility (Wang et al., 2014; Zhu and McBride, 2014). However, the encoding genes of other *C. hutchinsonii* T9SS components were difficult to delete for a long time. Recently, we optimized the screening medium of the *C. hutchinsonii* T9SS mutants, and successfully deleted *chu_0174* (*gldN*), which encodes a core component of T9SS. We found that *C. hutchinsonii* GldN not only participated in cellulose utilization and cell motility, but also played a crucial role in ion uptake (Gao et al., 2020). The functions of other components of *C. hutchinsonii* T9SS are still unclear.

In this study, the single deletion mutants of *chu_0029* (*sprA*) and *chu_2709* (*sprT*) were obtained using the modified complex medium with rich Ca^2+^ and Mg^2+^. It was found that *sprA* and *sprT* were both required for Ca^2+^ uptake. *sprA* and *sprT* were verified to encode essential components of *C. hutchinsonii* T9SS. Single deletion of *sprA* or *sprT* resulted in secretion defects of the majority of cell surface T9SS substrates based on the comparative proteomics analysis. Effects of deletion of *sprA* or *sprT* on Ca^2+^ acquisition, cellulose degradation, and cell motility were further studied.

## Materials and Methods

### Bacterial Strains, Plasmids, and Growth Conditions

Bacteria strains and plasmids used in this study are listed in Table 1. The primers used in this study are listed in Table 2. *Cytophaga hutchinsonii* ATCC 33406 was used as the wild type strain. Strains were cultivated in PY6 medium (6 g/liter peptone, 0.5 g/liter yeast extract, 4 g/liter glucose, pH 7.3), PYT medium (6 g/liter peptone, 0.5 g/liter yeast extract, 0.9 mM CaCl_2_, 0.8 mM MgSO_4_, 4 g/liter glucose, pH 7.3), Stanier medium (10 mM KNO_3_, 4.4 mM K_2_HPO_4_, 0.8 mM MgSO_4_, 0.07 mM FeCl_3_, 0.9 mM CaCl_2_, 2 g/liter glucose, pH 7.3) at 30°C with shaking at 160 rpm. PY2T media (2 g/liter peptone, 0.5 g/liter yeast extract, 0.9 mM CaCl_2_, 0.8 mM MgSO_4_, pH 7.3) with 2 g/liter glucose and 5 g/liter agar in soft agar or 0.5 g/liter glucose and 12 g/liter agar in hard agar were cultured in an incubator at 30°C. Plates with 10 g/liter agar (unless otherwise indicated) were used as the solid media which were cultured in an incubator at 30°C. *Escherichia coli* strains were cultured in Luria-Bertani medium at 37°C with shaking at 170 rpm or in an incubator at 37°C. Appropriate antibiotics were used at the following concentrations when needed: ampicillin, 100 μg/ml; erythromycin, 30 μg/ml; cefoxitin, 15 μg/ml.

**TABLE 1:** Strains and plasmids used in this study.

Strains or plasmids	Description*^*a*^*	Reference or source
*E. coli* strain		
DH5α	Strain used for gene cloning	TaKaRa
*C. hutchinsonii*		
ATCC 33406	Wild type	ATCC
Δ*sprA*	*chu_0029* deleted	This study
Δ*sprT*	*chu_2709* deleted	This study
Δ*0344*	*chu_0344* deleted	Wang et al. (2014)
Δ1336	*chu_1336* deleted	This study
Δ*0052*	*chu_0052* deleted	This study
Plasmids		
pCFX	Gene deletion template plasmid carrying *cfxA* flanked by two MCS; Ap^r^ (Cfx^r^), derived from pCFXSK with removal of the FRT sites.	This study
pCFXSK	Unmarked deletion template plasmid carrying *cfxA* flanked by two MCS; Ap^r^ (Cfx^r^)	Wang et al. (2014)
pSKSO8TG	Used for complementation of the mutant in *C. hutchinsonii* with *oriC*, Ap^r^ (Em^r^)	Wang et al. (2014)
pHB2709	Plasmid constructed from pSKSO8TG for complementation of Δ*sprT*; Ap^*r*^ (Em^*r*^)	This study

**^*a*^*MCS, multiple cloning site(s). FRT, FLP recombination target; Ap^r^, ampicillin resistance; Cfx^r^, cefoxitin resistance; Em^r^, erythromycin resistance. Phenotypes in parentheses are expressed in *C. hutchinsonii*, and phenotypes not in parentheses are expressed in *E. coli*.*

**TABLE 2:** Sequences of primers used in this study.

Primer	Sequence
0029H1F	CAGGGATCCAGATGGCTCGTATTATTCAT
0029H1R	AAACATATGGTCATACAACTCCGAAAATAC
0029H2F	TCAGTCGACCACTGCTGCCAAGTCAATTTAC
0029H2R	AAACGAGCTCGTAACCCGCTGCGAAATATT
0029UF	CATACTACCGTTATTCGGAGTT
0029UR	CACGCGTAATGTTAGGTGCTTC
2709H1F	ATAGGATCCAGGCCACGCAGATGCATTAG
2709H1R	CCTGGTACCTAGACAGGGGAAACACTTG
2709H2F	CGCGTCGACAAAACAACGGAAGTCAAC
2709H2R	GGGCATGCGTAAAAGACTCAAACGATG
2709UF	GCTTGGCTCCGTAATGAAAG
2709UR	CCACCGTAGGAAGCAGTCTC
C2709F	ACAGTCGACGTAGGGATTGACATTGCAGAAG
C2709R	ATAGAGCTCTTCTGTCTATAATCCACTTTTCT
CFXR	CTACAGCTGATATATGCGCAAC
QCFRT1F	TTAGTTGGCGCGCAATCAGTTCTTTAGCGATTA
QCFRT1R	TGATTGCGCGCCAACTAAGGAGGATATTCATATGGACCA
QCFRT2F	GAAGCAGCTCCAGCCTACACGTCG
QCFRT2R	CGACGTGTAGGCTGGAGCTGCTTCATTTAAATGGCGCGC TTTAAGATTT
16S-F	AAGGGTGAAACTCAAAGGA
16S-R	CTCGCTGGCAACTAAAGAT
q0052F	GGTGCGTATCGGTGAGTGG
q0052R	TATGCTCCCGTAGGACTTG
0028F	GCCTACATAGAAGGAAAATTAGCAC
0028R	GCGTAAATGAATTTGAGTTGCCT
0029F	ATTTTCTACCGTTCTAATAATGGGC
0029R	CATCACTCGTTAAGTTCGC
0030F	AAAATACAGCAAAGATCACGAATGG
0030R	GGCGGCTGCATCTAATAAAGCATC

### Genetic Constructs

Deletion of *chu_0029* or *chu_2709* was constructed by the homologous recombination as previously described (Wang et al., 2014). Briefly, a fragment containing the first 1846 bp of *chu_0029*, referred to as 0029H1, was amplified from the genome of *C. hutchinsonii* using primers 0029HIF and 0029H1R. 0029H1 was digested with BamHI and NdeI and ligated into the plasmid pCFX digested with the same enzymes to generate pCFX0029H1F. A fragment including the last 1858 bp of *chu_0029*, referred to as 0029H2, was amplified from the genome of *C. hutchinsonii* with primers 0029H2F and 0029H2R, digested with SalI and SacI, and then was ligated into the corresponding sites of pCFX0029H1F digested with the same enzymes. The gene-targeting cassette was amplified by PCR with primers 0029H1F and 00029H2R, then was purified using a Cycle Pure kit (Omega, Norcross, GA, United States). The electroporation procedure was performed as previously described (Wang et al., 2014). The transformants were cultivated on PYT plates with cefoxitin at 30°C for 10 to 15 days. The selected transformants were verified by PCR with primers 0029UF/0029UR and 0029UF/CFXR, and the PCR products were then verified by sequencing. The in-frame deletion of *chu_2709* was constructed using the same plasmid and method using the oligonucleotides specified in Table 2. The plasmid of pCFX was modified from pCFXSK with the removal of the two FRT sites. Reverse PCR was performed using pCFXSK as the template with primers QCFRT1F and QCFRT1R. The PCR product was digest with Dpn I to remove the template. Then the DNA was transformed into the *E. coli* DH5α. The plasmid was verified by sequencing to confirm the removal of the first FRT site. The removal of the second FRT site was constructed using the same method with primers QCFRT2F and QCFRT2R listed in Table 2. The map of pCFX is shown in Supplementary Figure 1.

### Complementation of the Δ*2709*

Δ*2709* was complemented as previously described using the replicative plasmid pSKSO8TG (Gao et al., 2020). Briefly, a fragment spanning *chu_2709*, containing 500 bp upstream of the start codon and 60 bp downstream of the stop codon, was amplified with primers C2709F and C2709R. The fragment was digested with SacI and SalI, then ligated into the linearized pSKSO8TG plasmid digested with the same enzymes to generate pHB2709. The plasmid was electroporated into Δ*2709*. PYT plates with cefoxitin and erythromycin were used to select the transformants. The complemented strain of Δ*2709* with pHB2709 was referred as C2709.

### Growth Analysis in Different Cultures

For growth analyses with different carbon sources, cells of *C. hutchinsonii* were precultured to mid-exponential phase using PYT medium. Cells were collected by centrifugation and washed with PY6 medium without carbon source, then adjusted to optical density at 600 nm (OD_600_) 1.0 for inoculation. In the case of glucose as the sole carbon source, the growth status was detected by the Bioscreen C analyzer (Oy growth curves Ab Ltd., Finland). Cells of the wild type, Δ*sprA* mutant, Δ*sprT* mutant were inoculated into 200 μl of PY6, PY6 with 0.8 mM MgSO_4_, PY6 with 0.9 mM CaCl_2_, Stanier, Stanier without the addition of CaCl_2_, Stanier with a reduced concentration of MgSO_4_ (from 0.8 mM to 0.1 mM), and PYT medium with an inoculum concentration of 3% (vol/vol) in sample plate. The sample plate was cultured at 30°C with shaking at medium speed. The growth status was monitored by the absorbance at 600 nm per 3 h. When Avicel and regenerated amorphous cellulose (RAC) were used as the sole carbon source, cellular protein concentration indicating the growth status was detected at set intervals as previously described (Gao et al., 2020). When determining the filter paper degradation ability, equivalent amounts of mid-exponential phase cells were spotted on Whatman filter paper overlaid on Stanier plates. The plates were incubated at 30°C in the incubator, and the utilization of filter papers was recorded at set intervals by a Canon camera.

### Detection of the Whole Cell and Intracellular Ion Content

The whole cell and intracellular concentrations of Mg^2+^ and Ca^2+^ were determined as described previously with some modification (Si et al., 2017a; Gao et al., 2020). Strains of the wild type, Δ*sprA* and Δ*sprT* mutants were cultured in 150 ml PY6 medium to mid-log phase. Cells were collected and washed twice with 10 ml 50 mM PIPES buffer. The cell pellets were resuspended in 6 ml PIPES buffer, and cells were broken by ultrasonication, as the whole cell samples. The cell debris was removed by centrifugation at 17,000 × *g* for 20 min at 4°C, and the supernatant was collected as the intracellular sample. The protein concentration was detected by the Bradford method following the manufacturer’s instructions. Each sample was diluted 25-fold in 1% (vol/vol) superior grade nitric acid to a total volume of 25 ml. The concentrations of Mg^2+^ and Ca^2+^ were detected by an inductively coupled plasma mass spectrometry analyzer (ICP-MS; NexION-1000G; PerkinElmer). The results were corrected using the appropriate buffers for reference and dilution factors. Triplicate cultures of each strain were analyzed during a single experiment, and the experiments were repeated at least three times.

### Observation of Colony Spreading and Microscopic Observation of Individual Cell Motility

Colony spreading was observed as previously described (Gao et al., 2020). Briefly, cells of the wild type and mutants were precultured in PYT medium to mid-exponential phase. Equivalent amounts of cells were spotted on PY2T soft agar. 100 μl of 100-fold diluted cells was evenly spread over the entire surface of PY2T hard agar. The plates were incubated at 30°C for about 4–10 days. Soft and hard agar spreading were recorded by a Canon camera and an IX51 phase contrast microscope (Olympus, Tokyo, Japan), respectively. Individual cell gliding motility over a glass surface was observed as previously described (Ji et al., 2013; Gao et al., 2020). Strains were precultured on PY2T plates with 2.0 g/L glucose at 30°C for about 4 days. Tunnel slides were prepared as previously described (Uenoyama et al., 2004). Cells suspended in TC buffer (10 mM Tris, 8 mM CaCl_2_, pH 7.3) were introduced into the tunnel slides, incubated for 5 min. The gliding motility of individual cells adhered to the glass cover slip was observed and recorded using an Olympus phase-contrast microscope with a heated stage at 30°C.

### Cellulase Activity Assays

The cellulase (endoglucanase) activity was detected as previously described (Ji et al., 2012; Gao et al., 2020). Briefly, cells were precultured in Stanier medium to mid-exponential phase. CMC-Na was used as the substrate to determine cellulase activity. For intact cell (cell surface) samples, cell pellets were washed and resuspended with Na_2_HPO_4_-KH_2_PO_4_ buffer (100 mM, pH 6.8). For cell extract samples, cell pellets were washed with Na_2_HPO_4_-KH_2_PO_4_ buffer, then resuspended with Na_2_HPO_4_-KH_2_PO_4_ buffer containing 2% (vol/vol) Triton X-100 and 0.5 mg/ml PMSF. The samples were incubated at 4°C for about 4 h. A mixture of 500 μl of resuspended cell samples and 500 μl of 1% (wt/vol) sodium carboxymethyl cellulose (CMC-Na) in distilled water was incubated for 30 min at 30°C. The reducing ends were measured using 3,5-dinitrosalicylic acid as previously described (Ji et al., 2012). The protein concentration was determined as described by Bradford (1976). The cell extract cellulase activity subtracted the cell surface cellulase activity is considered to be the intracellular cellulase activity.

### Assay of Bacterial Adhesion to Cellulose

Relative bacterial adhesion to Avicel PH-101 was measured by the turbidity-based method as described by Ji et al. (2012). Briefly, strains cultured in PYT medium were harvested by centrifugation at 5,100 × *g* for 5 min, washed with phosphate buffered saline (137 mM NaCl, 2.7 mM KCl, 10 mM Na_2_HPO_4_, 2 mM KH_2_PO_4_, pH 7.4), and resuspended with the same buffer to an optical density at 600 nm of 1.0. A 3.5 ml cell suspension was thoroughly mixed with 0.5 ml 10% (wt/vol) autoclaved Avicel PH101 for 10 min in room temperature. The mixture was left to stand in an incubator of 30°C for 1 h to allow cellulose, which adhered to bacterial cells, to settle. The optical density of the supernatant at 600 nm (OD_600_) was measured. The cell adhesion percentage was calculated using the following equation:

% adhesion = (1 − OD_600_) × 100

All of the measurements were carried out in triplicate.

### Detection of Cell Arrangement on Cellulose Fiber by Scanning Electron Microscopy

Cell arrangement on cellulose fiber was detected as previously described with some modifications (Xie et al., 2007). Briefly, strains were pre-cultured in Stanier medium to mid-log phase, cells were concentrated five times, and equivalent amounts of cells were incubated on Whatman No. 1 fiber paper on Stanier agar at 30°C for 48 h (the filter paper begins to turn slightly yellow). Samples were fixed with 2.5% glutaraldehyde in PBS buffer (pH 7.3) at 4°C for 12 h. Fixed cells were washed twice using PBS buffer, dehydrated through a graded series of ethanol (from 30%, 50%, 70%, 90%, 100%, and 100%, 15 min each time), and dried in a glass desiccator. Samples were processed according to a standard procedure and viewed by a SEM with a JEOL JSM-7600F field emission scanning electron microscope.

### Cell Fractionation and Western Blot Analysis

Outer membrane proteins were extracted as previously described (Wang S. et al., 2017; Gao et al., 2020). Briefly, strains were cultivated in Stanier medium to OD 0.6, and cells of equal biomass were harvested. The cell pellet was washed once with 50 mM piperazine-*N*,*N*’-bis (2-ethanesulfonic acid) (PIPES) buffer (pH 6.8), then resuspended with PIPES buffer with 0.5 M NaCl, and incubated at 4°C for 30 min with shaking at 150 rpm. Cells were removed by centrifugation at 12,000 × *g* for 10 min at 4°C, and the supernatant containing the buffer-washed proteins was ultracentrifuged (100,000 × *g*, 30 min, 4°C). The sediment was resuspended in PIPES buffer as outer membrane proteins. Extracellular proteins were isolated as described by Wang et al. (2014). Strains were cultured in PYT medium to OD 0.6, the culture was centrifuged at 5,000 × *g* for 10 min at 4°C, the supernatant was filtered through a 0.22-μm-pore-size polyvinylidene difluoride (PVDF) filter. The cell-free supernatant was concentrated using Amicon 10-kDa Ultra-15 centrifugal filter units (Millipore, MA, United States). Periplasmic proteins were extracted as described by Soares et al. (2003) with some modification. Strains were cultured in PYT or Stanier medium to OD 0.6, cells were collected by centrifugation at 5,000 × *g* for 10 min at 4°C. The cell pellets were resuspended by 0.3 M Tris-HCl pH 8.0 containing 20% sucrose and 1 mM EDTA, and incubated on ice for 15 min. Cells were collected by centrifugation at 7,600 × *g* for 10 min at 4°C. The cell pellets were resuspended vigorously by cold ddH_2_O, and incubated on ice for 15 min. The supernatant was collected by centrifugation at 12,000 × *g* for 10 min at 4°C, and was concentrated using Amicon 10-kDa Ultra-15 centrifugal filter units (Millipore, MA, United States). The protein concentrations were measured by the Braford method according to the manufacturer’s instruction. Outer membrane proteins with equal biomass were separated by sodium dodecyl sulfate-polyacrylamide gel electrophoresis (SDS-PAGE), then stained by Coomassie brilliant blue R-250. Differential bands between the WT and mutants were identified by mass spectrometry. Western blot was performed as previously described (Wang et al., 2014). Extracellular proteins, outer membrane proteins, and periplasmic proteins with equal biomass were separated by SDS-PAGE, and then transferred onto a PVDF membrane. For detection of CHU_0344 and CHU_0052, antibodies were the same as previously reported (Gao et al., 2020). The antibody against Cel9A was prepared by heterologous expression of Cel9A in *E. coli* BL21 (DE3). The purified Cel9A was used to immunize the rabbit to generate antibody (unpublished data).

### RT-PCR and Real-Time Quantitative PCR (qPCR) Analysis

Strains were cultured in Stanier medium to mid-log phase. Cell pellets of 4 ml of the culture were collected. RNA extraction was performed as previously described (Wang S. et al., 2017; Guan et al., 2018). The cDNAs of the wild type and Δ*sprA* mutant were used as the templates to perform RT-PCR with primers listed in Table 2 (0028F, 0028R, 0029F, 0029R, 0030F, 0030R). qPCR was performed on a LightCycler480 System with SYBR Green Premix Pro Taq HS qPCR Kit (Accurate Biotechnology, Hunan). The relative quantitation/comparative threshold cycle (ΔΔ*C*_*T*_) method (Livak and Schmittgen, 2001) was used to analyze the data, and the data was normalized to an endogenous control (16S rRNA gene). Three biological repeats were set for all assays.

### Cell Surface Shaving and LC-MS/MS

The protocol for cell surface trypsinization was adapted from Remus et al. (2013) with some modification. Strains were cultured in Stanier medium to OD 0.6. Cells were collected by centrifugation at 5,000 × *g* for 10 min at 4°C. Cell pellets were washed twice with 20 ml 20 mM Tris-HCl (pH 7.6) buffer containing 150 mM NaCl, 1 M xylose, 20 mM CaCl_2_, and 5 mM dithiothreitol (DTT), and subsequently were resuspended in 5 ml of the same buffer followed by the addition of 20 μg spectrometry-grade trypsin (T6567; Sigma-Aldrich, St. Louis, MO, United States). Samples were incubated at 30°C with shaking at 60 rpm for 1 h, and centrifuged at 5,000 × *g* for 10 min at 4°C to collect the supernatants. The tryptic peptide containing supernatants were filtered through a cellulose-acetate filter (0.22 μm pore size, 25 mm diameter, Sigma-Aldrich). The samples were freeze-dried to concentrate and stored at −80°C prior to LC-MS/MS. The samples were sent to the Beijing Genomics Institute for LC-MS/MS analysis.

### Disk Diffusion Susceptibility Test

Disk diffusion susceptibility was performed as described by Bai et al. (2017) with some modifications. Strains were cultured in PY6 medium or PYT medium to mid-log phase, and adjusted to OD_600_ of 1.0, and then 100 μl of the resuspended cells was evenly spread over the entire surface of PY6 or PYT agar plates. An 8-mm paper disk was placed on the plate and 3 μl of antimicrobial agent was added on the central of the paper. Plates were incubated for 3–6 days at 30°C, and the inhibition zone diameters were determined. The antimicrobial agents tested were hydrogen peroxide (2%), dithiothreitol (2 M), sodium dodecyl sulfate (10%), crystal violet (1%), gentamicin (20 μg/ml), and kanamycin (100 μg/ml).

### Extraction of LPS

Strains were cultured in PY6 or PYT medium to mid-log phase. Cell pellets of 4 ml of the culture were collected. The LPS was extracted as described by Davis and Goldberg (2012). First, prepare 2x SDS buffer (50 ml solution of 4% β-mercaptoethanol, 4% SDS and 20% glycerol in 0.1 M Tris-HCl, pH 6.8, add a pinch of bromophenol blue to dye the solution). Make a 1x SDS-buffer by diluting 2x SDS buffer 1:1 in sterile distilled H_2_O. Resuspend the cell pellets with 200 μl of 1x SDS-buffer. Ensure that the pellets were completely resuspended by pipetting the solution up and down slowly. Boil the suspended bacteria in a water bath for 15 min, then allow the solution to cool at room temperature for 15 min. Add 5 μl of DNase I (10 mg/ml) and RNase (10 mg/ml) solutions, then incubate the samples at 37°C for 30 min. Add 10 μl of Proteinase K (10 mg/ml) and incubate the samples at 59°C for 3 h. Add 200 μl of ice-cold Tris-saturated phenol to each sample, and vortex each sample for 10 s. Incubate the samples at 65°C for 15 min. After incubating cool to room temperature, then add 1 ml of room-temperature diethyl ether to each sample and vortex for 10 s. Centrifuge the samples at 12,000 × *g* for 20 min. Remove the upper, clear layer, and collect the bottom blue layer. Re-extract the samples by repeating the addition of room-temperature diethyl ether, and centrifugation. Add 200 μl of 2x SDS-buffer to each of the extracted sample before separating by Tris-Tricine-SDS-PAGE. The bands were visualized by silver-staining using LPS staining kit (Beyotime, P0017S).

### Statistical Analysis

Statistical analysis was performed using a Student’s *t*-test analysis. Three biological replicates were undertaken for each analysis. Reported results and errors are means and standard deviations, respectively, for these replicates.

## Results

### Individual Deletion of *chu_0029* and *chu_2709*

Protein blast revealed that CHU_0029 is similar in sequence to *F. johnsoniae* SprA (32% identity over 2380 amino acids), and CHU_2709 is similar in sequence to *F. johnsoniae* SprT (27% identity over 225 amino acids). SprA and SprT are important components of T9SS in *F. johnsoniae* (Sato et al., 2010; Lauber et al., 2018). To verify whether CHU_0029 and CHU_2709 are components of *C. hutchinsonii* T9SS, their encoding genes were deleted by homologous recombination using PYT medium as previously reported (Gao et al., 2020). The deletion processes were shown in Supplementary Figure 2. The single deletion mutants of *chu_0029* and *chu_2709* could not be obtained using normal medium (PY6), whereas could be obtained using normal medium with addition of Ca^2+^ and Mg^2+^ (PYT), indicating that CHU_0029 and CHU_2709 may be involved in the uptake of trace amounts of Ca^2+^ and Mg^2+^. The selected transformants were verified by PCR, and all had the expected band sizes (see Supplementary Figure 3). Hereafter, *chu_0029* and *chu_2709* were designated *sprA* and *sprT*, respectively.

### *sprA* and *sprT* Are Involved in the Uptake of Trace Amount of Ca^2+^

**FIGURE 1:**
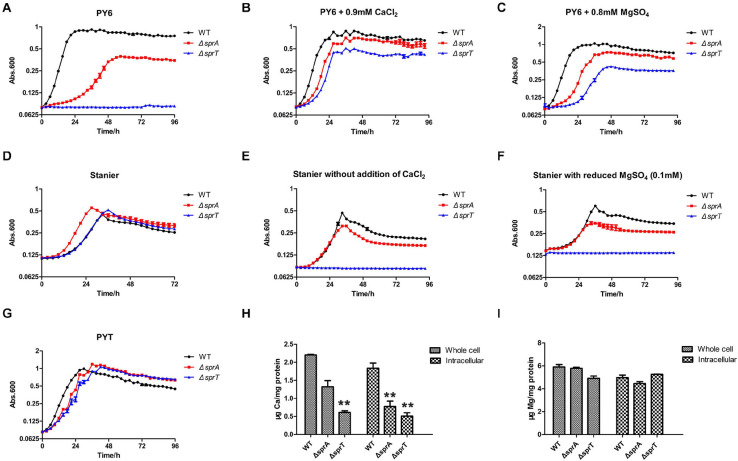
SprA and SprT are involved in the uptake of trace amount of Ca^2+^. Growth curves of the wild type, Δ*sprA* mutant, and Δ*sprT* mutant in different media. **(A)** PY6, **(B)** PY6 with CaCl_2_ (0.9 mM), **(C)** PY6 with MgSO_4_ (0.8 mM), **(D)** Stanier, **(E)** Stanier without the addition of CaCl_2_, **(F)** Stanier with a reduced concentration of MgSO_4_ (0.1 mM), **(G)** PYT. Growth status was monitored by the absorbance at 600 nm. Abs, absorbance. Whole cell and intracellular concentrations of Ca^2+^ **(H)** and Mg^2+^ **(I)** were detected by ICP-MS. Strains were cultured in PY6 medium to the mid-log phase. **p < 0.01. WT, wild type strain; Δ*sprA, chu_0029* deletion mutant; Δ*sprT, chu_2709* deletion mutant. Three biological replicates were set, and the mean values and SDs are shown.

Above work showed that SprA and SprT may be involved in the uptake of trace amounts of Ca^2+^ and Mg^2+^. In order to verify this possibility, the growth curves of the mutants in different media were measured. As shown in Figure 1A, the Δ*sprA* and Δ*sprT* mutants all grew poorly in PY6 medium, especially the Δ*sprT* mutant, with a long lag phase and reduced maximum biomass compared with the wild type (Figure 1A). However, the addition of CaCl_2_ (0.9 mM) and MgSO_4_ (0.8 mM), respectively, to PY6 medium could significantly shorten the lag phase and increase the maximum biomass of the mutants (Figures 1B,C). Moreover, although the Δ*sprA* and Δ*sprT* mutants grew as well as the wild type in Stanier (Figure 1D), they all showed growth defects in Stanier without the addition of CaCl_2_ or with reduced addition of MgSO_4_ (from 0.8 mM to 0.1 mM). In these two media, the Δ*sprA* mutant grew with obviously reduced maximum biomass, and the Δ*sprT* mutant did not grow at all during the cultivation of 4 days (Figures 1E,F). In PYT medium, the Δ*sprA* and Δ*sprT* mutants could grow almost as well as the wild type (Figure 1G). In order to further study whether SprA and SprT are involved in the assimilation of Ca^2+^ and Mg^2+^, the whole cell and intracellular concentrations of Ca^2+^ and Mg^2+^ of the wild type, Δ*sprA* and Δ*sprT* mutants were determined using inductively coupled plasma mass spectrometry (ICP-MS). The whole cell concentrations of Ca^2+^ of the Δ*sprA* and Δ*sprT* mutants were decreased by 40% and 72%, respectively, compared with the wild type. Meanwhile, the intracellular concentrations of Ca^2+^ of the Δ*sprA* and Δ*sprT* mutants were reduced by 58% and 72%, respectively, compared with the wild type (Figure 1H). The whole cell concentrations of Mg^2+^ of the Δ*sprA* and Δ*sprT* mutants were decreased by 2% and 15%, respectively, compared with the wild type. Additionally, the intracellular concentration of Mg^2+^ was decreased by 10% in the Δ*sprA* mutant. However, the intracellular concentration of Mg^2+^ of the Δ*sprT* mutant was equivalent to that of the wild type (Figure 1I). These results demonstrated that SprA and SprT are both involved in the uptake of trace amount of Ca^2+^. The Δ*sprA* and Δ*sprT* mutants were cultured using PYT medium from then on unless specially stated.

### The Δ*sprA* and Δ*sprT* Mutants Are Defective in Cellulose Utilization

**FIGURE 2:**
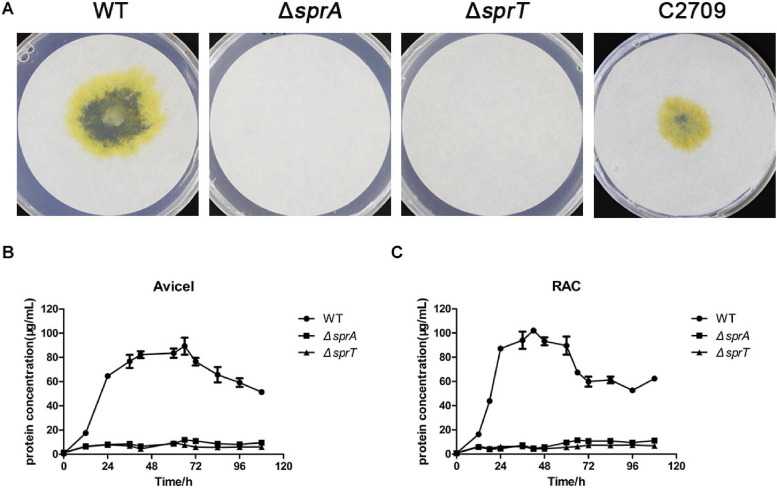
Cellulose utilization ability of the wild type, Δ*sprA* mutant, and Δ*sprT* mutant. **(A)** Filter paper degradation ability. Strains were precultured in Stanier medium. Equivalent amounts (3 μl) of cells were spotted on the Whatman filter paper on Stanier agar, followed by incubation for 15 days at 30°C. This assay was performed in triplicate using three independent transformants (with the same results), and one representative result is shown. The utilization ability of **(B)** 0.2% Avicel and **(C)** 0.2% RAC. The mean values and SDs from at least three replicates are shown. WT, wild type strain; Δ*sprA*, *chu_0029* deletion mutant; Δ*sprT*, *chu_2709* deletion mutant; C2709, the Δ*sprT* mutant complemented with pHB2709.

In order to investigate whether the deletion of *sprA* or *sprT* affected the degradation of cellulose, we determined the cellulose utilization ability of the Δ*sprA* and Δ*sprT* mutants. Wild type grew rapidly on plate of Stanier agar with Whatman No. 1 filter paper as the sole carbon and energy source, whereas the Δ*sprA* and Δ*sprT* mutants failed to degrade filter paper even after incubation for 15 days (Figure 2A). Moreover, cells of the Δ*sprA* and Δ*sprT* mutants failed to utilize 0.2% Avicel (Figure 2B) and 0.2% regenerated amorphous cellulose (RAC) (Figure 2C) in liquid Stanier medium. These results demonstrated that SprA and SprT are essential for cellulose degradation. Complementation of the Δ*sprT* mutant with pHB2709, which carries *sprT*, restored the ability of the Δ*sprT* mutant to digest filter paper (Figure 2A), confirming the role of SprT in cellulose utilization.

*sprA* contains 7143 bp, which is too large to complement the Δ*sprA* mutant. RT-PCR was used to determine the transcription of the adjacent genes of *sprA*. As shown in Supplementary Figure 4, *chu_0028* and *chu_0030* could be normally expressed in the Δ*sprA* mutant as the wild type did, indicating that the phenotypes of the Δ*sprA* mutant was due to the absence of *sprA*.

### The Δ*sprA* and Δ*sprT* Mutants Had Decreased Cell Surface Cellulase Activities and Adhesion Abilities to Cellulose Fiber

**FIGURE 3:**
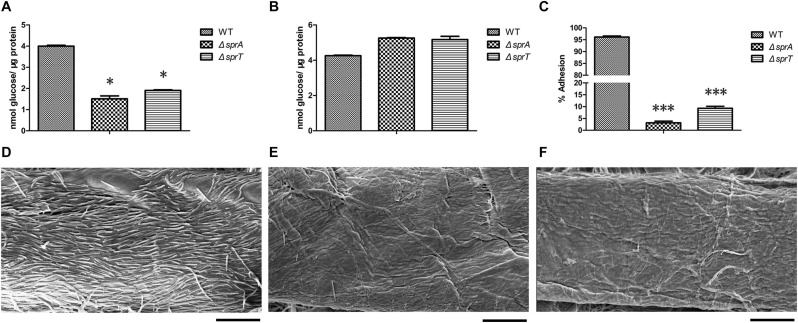
The cellulase activity and cellulose adhesion ability of the wild type, Δ*sprA* mutant, and Δ*sprT* mutant. The cell surface **(A)** and intracellular **(B)** cellulase activity were determined. Strains were precultured in Stanier medium to mid-log phase. CMC-Na was used as the substrate, and the concentrations of the reducing end were measured using the 3,5-dinitrosalicylic acid procedure. The data shown are the mean values and SDs from three independent experiments. *p < 0.05. **(C)** Cellulose adhesion percentage of the WT, Δ*sprA* mutant, and Δ*sprT* mutant cells. The mean values and SDs from at least three replicates are shown. ***p < 0.001. Colonization behavior of the WT **(D)**, Δ*sprA* mutant **(E)**, and Δ*sprT* mutant **(F)** cells on filter paper fiber. Equivalent amounts of cells were spotted on the Whatman filter paper, cultured for 2 days, then detected by SEM. Bars, 10 μm. Three biological replicates were set (with the same results), and one representative result is shown.

In *C. hutchinsonii*, there are 12 predicted endoglucanases containing conserved CTDs, which are supposed to be secreted, and then anchored on the cell surface by T9SS. These endoglucanases were abundant during cellulose degradation, especially CHU_1075 and CHU_1107, implying that they may play crucial roles in cellulose utilization (Taillefer et al., 2018). To investigate whether the deletion of *sprA* or *sprT* affected the secretion of these endoglucanases, the cell surface and intracellular cellulase activity were determined as described in the Section “Materials and Methods.” As shown in Figure 3A, the cell surface cellulase activities of the Δ*sprA* and Δ*sprT* mutants were decreased by 62% and 52%, respectively, compared with the wild type, whereas the intracellular cellulase activities of the Δ*sprA* and Δ*sprT* mutants were increased by 23% and 21%, respectively (Figure 3B). The result suggested the secretion defects of cell surface endoglucanases and the accumulation of them in the periplasmic space of the Δ*sprA* and Δ*sprT* mutants.

Direct contact of *C. hutchinsonii* cells with cellulose is needed in the process of cellulose degradation, and regular arrangement of *C. hutchinsonii* cells was detected along the cellulose fiber (Wilson, 2009; Zhu and McBride, 2017). The unique cellulose degradation mechanism of *C. hutchinsonii* suggested that the cellulose adhesion ability is crucial for cellulose degradation. In order to determine whether the deletion of *sprA* or *sprT* affects cellulose adhesion ability, the cell adhesion abilities to Avicel of the wild type, Δ*sprA* and Δ*sprT* mutants were measured as previously described. The cellulose adhesion percentage of the wild type cells was 96% (Figure 3C), whereas cells of the Δ*sprA* and Δ*sprT* mutants could hardly adhere to Avicel, the adhesion percentages were 3% and 9%, respectively (Figure 3C). Moreover, the cell colonization behavior of the wild type, Δ*sprA* and Δ*sprT* mutants on filter paper fiber was observed by scanning electron microscopy (SEM) as described in the Section “Materials and Methods.” As shown in Figure 3D, a large number of the wild type cells which regularly arranged along the filter paper fiber were detected. Notably, only a few cells of the Δ*sprA* and Δ*sprT* mutants were detected on the filter paper fiber (Figures 3E,F).

### Deletion of *sprA* or *sprT* Resulted in Defects in Cell Motility

**FIGURE 4:**
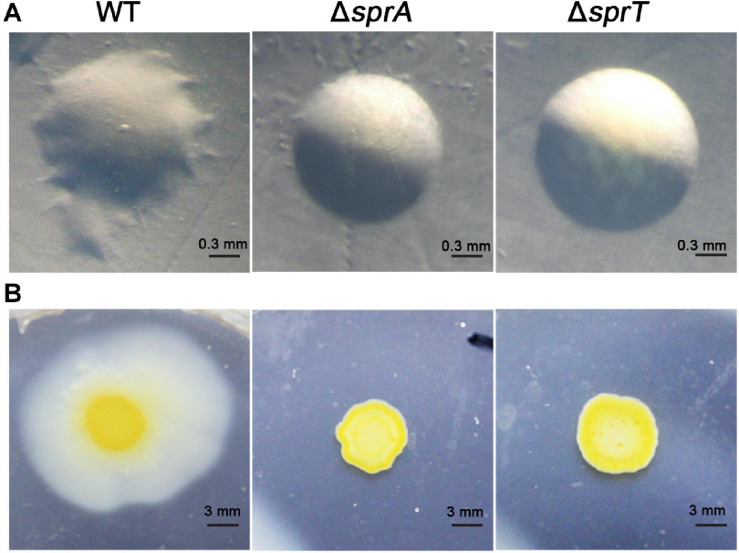
Colony spreading of the wild type, Δ*sprA* mutant, and Δ*sprT* mutant. **(A)** Colony spreading on PY2T hard agar at 30°C for 4 days. **(B)** Colony spreading on PY2T soft agar at 30°C for 10 days. WT, wild type; Δ*sprA*, *chu_0029* deletion mutant; Δ*sprT*, *chu_2709* deletion mutant. The colony spreading assays were performed in triplicate using three independent transformants (with the same results), and one representative result is shown.

Rapid gliding over solid surfaces without flagella or type IV pili is another feature of *C. hutchinsonii*. It is speculated that gliding motility is crucial for *C. hutchinsonii* to efficiently digest cellulose (Stanier, 1942; Xie et al., 2007; Zhu and McBride, 2017). In *F. johnsoniae*, gliding motility relies on the rapid movement of SprB and RemA, which are secreted by the T9SS to the cell surface (McBride, 2019). To investigate the effect of deletion of *sprA* or *sprT* on cell motility, colony spreading of the wild type, Δ*sprA* and Δ*sprT* mutants was studied on PY2T plates. Cells of the wild type formed spreading colonies on hard agar and soft agar, whereas cells of the Δ*sprA* and Δ*sprT* mutants lost the ability to form spreading colonies on both hard agar and soft agar (Figure 4). Further, individual cell motility of the wild type, Δ*sprA* and Δ*sprT* mutants was determined. It was found that individual cells of the wild type could vigorously glide over the glass surface (Supplementary Movie 1). However, a few cells of the Δ*sprA* mutant were detected on the glass surface, suggesting that individual cells of the Δ*sprA* mutant had decreased adhesion ability to the glass surface. Moreover, the adhered cells could not glide (Supplementary Movie 2). Though cell adhesion ability to the glass surface of the Δ*sprT* mutant was not dramatically decreased, cells of the Δ*sprT* mutant also lost the ability to glide on the glass surface (Supplementary Movie 3).

### *sprA* and *sprT* Are Essential for the Secretion of Extracellular T9SS Substrate

**FIGURE 5:**
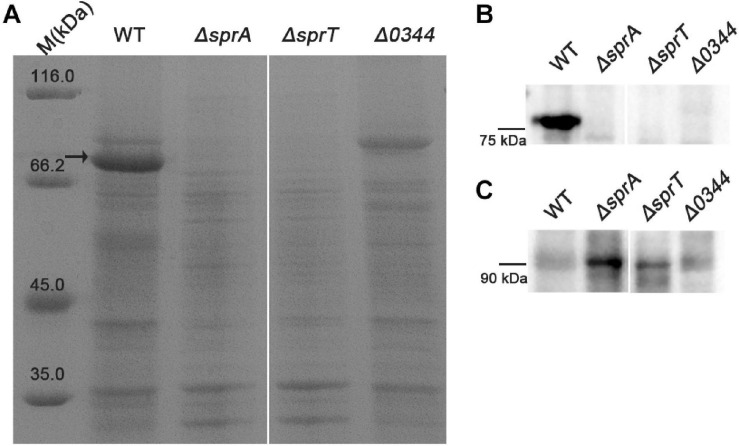
Secretion defects of CHU_0344 in the Δ*sprA* and Δ*sprT* mutants. **(A)** The extracellular protein profiles of the wild type, Δ*sprA* mutant, and Δ*sprT* mutant. Western blot determined the abundance of CHU_0344 in the extracellular proteins **(B)** and periplasmic proteins **(C)** extracted from the wild type, Δ*sprA* mutant, and Δ*sprT* mutant. Strains were cultured in PYT medium to OD 0.6, loading samples were normalized by equal biomass. The arrow indicates CHU_0344 identified by mass spectrometry. WT, wild type strain; Δ*sprA*, *chu_0029* deletion mutant; Δ*sprT, chu_2709* deletion mutant. Δ*0344*, *chu_0344* deletion mutant. All measurements were carried out in triplicate with the same results. The full size of SDS-PAGE gel and Western blot figure are shown in Supplementary Figures 5, 6.

As a portion of T9SS substrates could be released to the extracellular medium in *C. hutchinsonii* (Wang et al., 2014; Zhu and McBride, 2014), deletion of the encoding genes of T9SS would result in defect in secretion of extracellular T9SS substrates. To determine the effect of deletion of *sprA* or *sprT* on the secretion of extracellular T9SS substrates, the extracellular proteins of the wild type, Δ*sprA* and Δ*sprT* mutants were extracted as described in the Section “Materials and Methods,” and separated by SDS-PAGE. As shown in Figure 5A, the main extracellular protein was missing in the extracellular proteins extracted from the Δ*sprA* and Δ*sprT* mutants, which was identified to be CHU_0344 by mass spectrometry. CHU_0344 is the dominant extracellular protein and is verified to be secreted by T9SS (Gao et al., 2020). Further, a Western blot using an antibody against CHU_0344 detected abundant CHU_0344 in the extracellular proteins extracted from the wild type, but CHU_0344 was not detected in the extracellular proteins extracted from the Δ*sprA* and Δ*sprT* mutants (Figure 5B). Moreover, a protein band with a molecular mass similar to the full length of CHU_0344 with intact CTD was detected in the periplasmic proteins of the Δ*sprA* and Δ*sprT* mutants (Figure 5C). These results indicated the secretion defects of CHU_0344 in the Δ*sprA* and Δ*sprT* mutants, suggesting that SprA and SprT are involved in the secretion of extracellular T9SS substrates.

### Outer Membrane Protein Profiles Were Obviously Changed in the Δ*sprA* and Δ*sprT* Mutants

**FIGURE 6:**
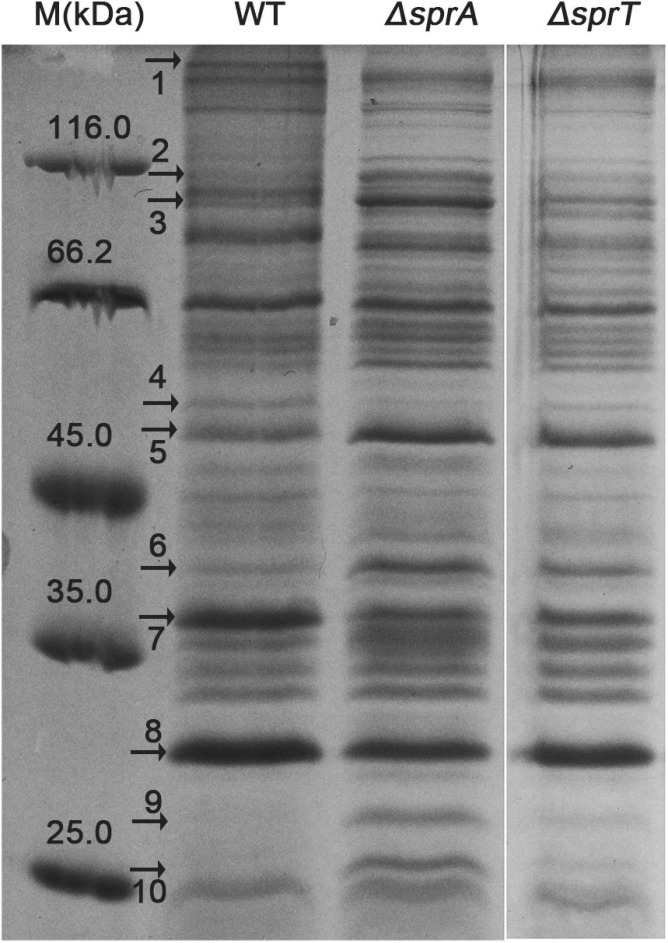
SDS-PAGE of the outer membrane proteins of the wild type, Δ*sprA* mutant, and Δ*sprT* mutant. Strains were cultured in Stanier medium to mid-log phase. Loading samples were normalized by equal biomass. Differential protein bands between the WT, Δ*sprA* mutant, and Δ*sprT* mutant were marked by black arrows and identified by mass spectrometry. WT, wild type strain; Δ*sprA*, *chu_0029* deletion mutant; Δ*sprT*, *chu_2709* deletion mutant. This measurement was carried out in triplicate with the same results. The full size of the SDS-PAGE gel is shown in Supplementary Figure 7.

Because direct contact of *C. hutchinsonii* cells with cellulose is necessary for cellulose degradation, outer membrane proteins are speculated to play significant roles in the process of cellulose utilization (Xie et al., 2007). As possible components of T9SS, SprA and SprT may participate in the translocation of T9SS substrates from the periplasmic space to the outer membrane. In order to examine the effect of deletion of *sprA* or *sprT* on the location of outer membrane proteins, the outer membrane proteins were extracted as previously described, and separated by SDS-PAGE (Gao et al., 2020). As shown in Figure 6, deletion of *sprA* or *sprT* obviously affected the outer membrane protein profiles, 10 proteins were changed in abundance compared with the wild type. The differential bands were identified by mass spectrometry, and the results were shown in Table 3. CHU_1075, belonging to the glycoside hydrolase family 8, is an endoglucanase with a conserved CTD, which was abundant during the cellulose degradation (Taillefer et al., 2018). CHU_1075 was absent on the outer membrane proteins of the Δ*sprA* and Δ*sprT* mutants, implying that SprA and SprT are essential for the secretion of CHU_1075. CHU_2807, which is involved in the acquisition of trace amounts of Ca^2+^ and Mg^2+^ (Gao et al., 2020), was decreased in abundance in the outer membrane proteins of the Δ*sprA* and Δ*sprT* mutants. Besides, CHU_0052, annotated as DegQ, was increased in abundance in the outer membrane proteins of the Δ*sprA* and Δ*sprT* mutants. CHU_0052 is a homologous protein of HtrA, which plays an important role in protein quality control (Boehm et al., 2018). In addition, seven proteins with unknown functions were differentially expressed in the Δ*sprA* and Δ*sprT* mutants. Functions of these proteins in *C. hutchinsonii* are under study.

**TABLE 3:** Identification of the differential outer membrane proteins between the wild type and T9SS mutants.

Band	Locus	MW (kDa)*^*a*^*	Predicted function	CTD	Abundance*^*b*^*
1	CHU_1075	274.2	GH8 endoglucanase	TIGR04183	_
2	CHU_1230	110.6	Zinc protease	_	↑
3	CHU_0192	88.1	Possible peptidyl-prolyl *cis*–*trans* isomerase	_	↑
4	CHU_2807	49.9	Outer membrane efflux protein	_	↓
5	CHU_0052	51.8	Serine protease	_	↑
6	CHU_3238	42.8	Conserved hypothetical protein	_	↑
7	CHU_3384	33.8	Hypothetical protein	_	↓
8	CHU_0007	27.4	Hypothetical protein	_	↓
9	CHU_3414	23.7	Outer membrane protein	_	↑
10	CHU_2253	24.8	Conserved hypothetical protein	_	↑

**^*a*^*MW, Molecular mass of the primary product of translation, including the predicted signal peptide. *^*b*^*Abundance change: – missing, ↑ increased in abundance, ↓ decreased in abundance.*

### Deletion of *sprA* or *sprT* Resulted in Significant Defects in Secretion of Cell Surface T9SS Substrates

It is speculated that T9SS substrates may play vital roles in cellulose degradation and cell motility in *C. hutchinsonii*, and the majority of them would be anchored on the cell surface (Veith et al., 2017). However, the SDS-PAGE of the outer membrane proteins of the wild type, Δ*sprA* and Δ*sprT* mutants did not show many distinguishable differences. It may be attributed to the low resolution of one-dimensional electrophoresis. Therefore, an alternative, non-gel-based approach was employed. To investigate changes in the cell surface proteins of the Δ*sprA* and Δ*sprT* mutants, cell surface shaving with trypsin and LC-MS/MS were used as described in the Section “Materials and Methods.” More than two hundred proteins in each sample derived from the wild type, Δ*sprA* and Δ*sprT* mutants were identified. 41 CTD proteins were detected on the cell surface of the wild type (Supplementary Data Sheet 1). Notably, 22 CTD proteins were absent and 10 CTD proteins were significantly decreased in abundance on the cell surface the Δ*sprA* mutant. Seven CTD proteins were missing and 13 CTD proteins were dramatically decreased in abundance on the cell surface the Δ*sprT* mutant (Supplementary Data Sheet 1). These CTD proteins are supposed to be transported across the outer membrane to the cell surface by the T9SS. These results indicated that SprA and SprT are essential for the cell surface localization of the majority of CTD proteins.

### Secretion Defects of Cel9A and the Increased Expression Level of *degQ* in the Δ*sprA* and Δ*sprT* Mutants

**FIGURE 7:**
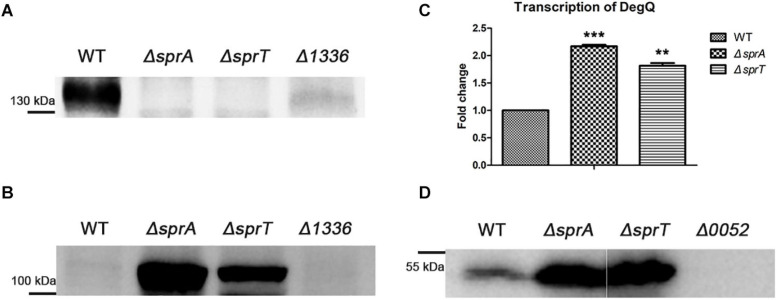
Secretion defects of Cel9A and the increased expression level of *degQ* in the Δ*sprA* and Δ*sprT* mutants. Western blot detected the abundance of Cel9A in the outer membrane proteins **(A)** and periplasmic proteins **(B)** using an antibody against Cel9A (the Δ*1336* mutant was used as a negative control). Strains were cultured in Stanier medium to OD 0.6. Loading samples were normalized by equal biomass. **(C)** qPCR detected the transcription levels of *degQ* in the wild type, Δ*sprA* mutant, Δ*sprT* mutant. Strains were cultured in Stanier medium to OD 0.6. The mean values and SDs from at least three replicates are shown. **p < 0.01, ***p < 0.001. **(D)** Western blot detected the abundance of DegQ in the periplasmic space of wild type, Δ*sprA* mutant, and Δ*sprT* mutant. Strains were cultured in Stanier medium to the mid-log phase (the Δ*0052* mutant was used as a negative control). The full size of the Western blot figure is shown in Supplementary Figure 8. WT, wild type strain; Δ*sprA*, *chu_0029* deletion mutant; Δ*sprT, chu_2709* deletion mutant. Δ*1336*, *chu_1336* deletion mutant; Δ*0052*, *chu_0052* deletion mutant. Loading samples were normalized by equal biomass. All measurements were carried out in triplicate with the same results.

CHU_1336 (Cel9A) is a family-9 endoglucanase with a conserved CTD and is predicted to be located on the cell surface (Zhu et al., 2016). Cel9A could efficiently hydrolyze carboxymethyl-cellulose and was proved to act as a non-processive enzyme with endo-cellulase activities (Louime et al., 2007). The above result indicated that the abundance of Cel9A was significantly decreased on the cell surface of the Δ*sprA* and Δ*sprT* mutants. To further verify the involvement of SprA and SprT in the secretion of Cel9A, a Western blot analysis using an antibody against Cel9A was performed. Cel9A was detected in the outer membrane proteins extracted from the wild type, but was absent in the outer membrane proteins extracted from the Δ*sprA* and Δ*sprT* mutants (Figure 7A). It was found that the mature Cel9A was supposed to be modified by an unknown mechanism with a molecular weight above 130 kDa (the theoretical molecular weight of the primary product of Cel9A is 105 kDa). The accumulation of Cel9A in the periplasmic space of the Δ*sprA* and Δ*sprT* mutants was detected (Figure 7B). These results indicated that SprA and SprT are involved in the secretion of Cel9A. Our results demonstrated that SprA and SprT participated in the secretion of the majority of T9SS substrates. Deletion of *sprA* or *sprT* resulted in the secretion defects of CTD proteins, which would be accumulated in the periplasmic space of the Δ*sprA* and Δ*sprT* mutants. To verify this, we determined the expression level of *chu_0052* (*degQ*). DegQ locates in periplasmic space, and is the main homologous protein of HtrA in *C. hutchinsonii* (Gao et al., 2020; Wang et al., 2020), which plays vital roles in degradation and refolding of the aberrant proteins. As shown in Figure 7C, the transcription level of *degQ* in the Δ*sprA* and Δ*sprT* mutants was 2.2 and 1.8 times of that of the wild type. A Western blot using an antibody against DegQ further demonstrated the obvious increased expression level of DegQ in the Δ*sprA* and Δ*sprT* mutants (Figure 7D). These results implied that a large number of T9SS substrates accumulated in the periplasmic space of the Δ*sprA* and Δ*sprT* mutants.

### The Increased Outer Membrane Permeability and Cell Sensitivity to Antibiotics of the Δ*sprA* and Δ*sprT* Mutants

In order to determine the stability and the barrier function of the outer membrane of the Δ*sprA* and Δ*sprT* mutants, strains were cultured in media with low concentrations of Mg^2+^ and Ca^2+^ (PY6) or high concentrations of Mg^2+^ and Ca^2+^ (PYT). The outer membrane permeability, oxidant-reductant sensitivity, and resistance to antibiotics of the Δ*sprA* and Δ*sprT* mutants were determined using crystal violet, sodium dodecyl sulfate, H_2_O_2_, dithiothreitol, gentamicin, and kanamycin as described in the Section “Materials and Methods.” The results of antimicrobial experiment showed that cells of the Δ*sprA* and Δ*sprT* mutants were more sensitive to antimicrobial agents, and cells of the Δ*sprA* and Δ*sprT* mutants cultured in PY6 medium or PYT medium had similar sensitivity to harmful compounds (Supplementary Figures 9, 10). Despite that the Δ*sprA* and Δ*sprT* mutants needed more time to grow on PY6 plates. It is speculated that these phenotypes might result from the absence of some outer membrane T9SS substrates. Previous study reported that the lipopolysaccharide (LPS) of the Gram-negative bacteria could bind divalent cations, such as Ca^2+^ and Mg^2+^. The presence of divalent cations, especially Mg^2+^, could strengthen the structure of LPS and increase the barrier function of the outer membrane (Murata et al., 2007; Herrmann et al., 2015; Smart et al., 2017; Simpson and Trent, 2019). The LPS was extracted from cells of the wild type, Δ*sprA* and Δ*sprT* mutants cultured in PY6 medium or PYT medium. The results indicated that the LPSs of the wild type, Δ*sprA* and Δ*sprT* mutants were almost identical, and low concentrations of Mg^2+^ and Ca^2+^ did affect the LPSs of the wild type, Δ*sprA* and Δ*sprT* mutants (Supplementary Figure 11). These results demonstrated that the growth defects of the Δ*sprA* and Δ*sprT* mutants in Ca^2+^- and Mg^2+^-deficient conditions were not caused by the outer membrane defects and changes in LPS structure.

## Discussion

*Cytophaga hutchinsonii* is a widely distributed bacterium, which could efficiently digest cellulose in a cell contact dependent mode (Xie et al., 2007). The cellulose degradation mechanism of *C*. *hutchinsonii* is unique and different from the well-studied two strategies: secretion of soluble cellulases or production of multiprotein cellulosome (Fontes and Gilbert, 2010; Wilson, 2011). T9SS is widely and exclusively distributed in the the phylum *Bacteroidetes*, and is essential for the secretion of proteins which are involved in pathogenicity, motility, and degradation of cellulose and chitin (Sato et al., 2010; Lasica et al., 2017; Veith et al., 2017). *C*. *hutchinsonii* has a full set of orthologs of T9SS components. Previous studies reported that single deletion of *chu_3237* (*porU*), *chu_0170* (*sprP*), or *chu_0174* (*gldN*) resulted in defects in cellulose degradation and motility (Wang et al., 2014; Zhu and McBride, 2014; Gao et al., 2020). However, the functions of other components of T9SS in *C*. *hutchinsonii* remain unknown. In this study, we characterized the functions of CHU_0029 (SprA) and CHU_2709 (SprT), which are similar in sequence to *F. johnsoniae* SprA and SprT, respectively. The secretion defects of CHU_0344, which is the main extracellular protein with a conserved CTD, in the Δ*sprA* and Δ*sprT* mutants indicated that SprA and SprT are involved in protein secretion. Through cell surface shaving and LC-MS/MS analysis, 41 CTD proteins could be detected on the cell surface of the wild type. Notably, single deletion of *sprA* and *sprT* resulted in disappearance or significant decrease of 32 and 20 CTD proteins on the cell surface, respectively (Supplementary Data Sheet 1). These results demonstrated that SprA and SprT are essential components of *C*. *hutchinsonii* T9SS. The majority of CTD proteins identified on the cell surface of the wild type have no clear functions and are only annotated as large proteins. The functions of these proteins need further dissection.

Recently, we reported that *C*. *hutchinsonii* GldN is essential for the acquisition of Ca^2+^ and Mg^2+^ under Ca^2+^- or Mg^2+^-deficient conditions (Gao et al., 2020). Here we found that single deletion of *sprA* or *sprT* also resulted in growth defects under Ca^2+^- or Mg^2+^-deficient conditions, especially *sprT*. The growth defects could be alleviated by the addition of Ca^2+^ or Mg^2+^. The significantly decreased intracellular concentrations of Ca^2+^ in the Δ*sprA* and Δ*sprT* mutants detected by ICP-MS further demonstrated that SprA and SprT participated in the acquisition of Ca^2+^. Whereas, the intracellular concentration of Mg^2+^ of the Δ*sprT* was almost consistent with that of the wild type, and it was only reduced by 10% in the Δ*sprA* mutant, compared with that of the wild type. The whole cell concentrations of Mg^2+^ of the Δ*sprA* and Δ*sprT* mutants were reduced by 2% and 15%, respectively, compared with that of the wild type. These results indicated that the uptake of Mg^2+^ was not significantly affected in the Δ*sprA* and Δ*sprT* mutants, and SprA and SprT play more important roles in assimilation of Ca^2+^ than that of Mg^2+^. Previous study reported that GldN is involved in the acquisition of Ca^2+^ and Mg^2+^ (Gao et al., 2020). Based on the results of our study, we speculated that different components of *C*. *hutchinsonii* T9SS exert different roles ion acquisition, and SprA, SprT, GldN are all involved in the acquisition of Ca^2+^. CHU_2807 is annotated as an outer membrane efflux protein with a TolC domain, which could form a trimeric channel structure. Our previous study reported that CHU_2807 is involved in the uptake of trace amounts of Ca^2+^ and Mg^2+^ (Gao et al., 2020). Here we found that the outer membrane abundance of CHU_2807 was obviously decreased in the Δ*sprA* and Δ*sprT* mutants. This could partly account for the growth defects of the Δ*sprA* and Δ*sprT* mutants in PY6 medium. Bacterial proteins with immunoglobulin-like (BIg-like) domain were reported to be involved in binding of Ca^2+^ (Dominguez et al., 2015). Scanning the genome of *C*. *hutchinsonii* revealed 10 CTD proteins with BIg-like domain (Supplementary Table 1), among which four proteins (CHU_0938, CHU_0939, CHU_1221, and CHU_2922) were absent or decreased in abundance on the cell surface of the Δ*sprA* and Δ*sprT* mutants. We speculated that these proteins would participate in Ca^2+^ acquisition. Genes of *chu_0938*, *chu_0939*, *chu_1221*, and *chu_2922* were individually deleted using homologous recombination method (*chu_0938* and *chu_0939* were deleted together, because they are in the same gene cluster). The transformants were cultured in PY6 medium or PYT medium. It was found that numbers of the transformants of these mutants cultured with PY6 medium were far less than that cultured with PYT medium. However, the growth curves of the mutants in PY6 medium did not show significant lag phase (unpublished data). These results indicated the redundant divalent cation transport system in *C. hutchinsonii*. Ca^2+^ plays important role in the physiology of bacteria (Gode-Potratz et al., 2010; Dominguez et al., 2015). However, the molecular mechanism of acquisition of Ca^2+^ in bacteria is elusive. There is only a report about the transportation of Ca^2+^ across the inner membrane through P-type ATPase (Gupta et al., 2017). The transport mechanism of Ca^2+^ across the outer membrane remains a mystery. Recently, it is reported that substrates of the type VI secretion system (T6SS) are involved in the transportation of iron, zinc, and manganese across the outer membrane (Lin et al., 2017; Si et al., 2017a, b). It is reasonable to speculate that certain T9SS substrates of *C*. *hutchinsonii* may be involved in the acquisition of Ca^2+^. These results suggested that there is a link between the T9SS and Ca^2+^acquisition system, and SprA and SprT are essential for this process.

Our results showed that SprA and SprT are involved in multiple physiological processes in *C*. *hutchinsonii*, including cellulose utilization, cellulose adhesion, and cell motility. Single deletion of *sprA* or *sprT* resulted in complete defects in degradation of crystalline and amorphous cellulose. In *C*. *hutchinsonii*, there are 12 predicted endoglucanases with conserved CTDs, which are supposed to be secreted from the periplasmic space to the cell surface by T9SS (Taillefer et al., 2018). Western blot using an antibody against Cel9A, which is a cell surface non-processive endoglucanase with CTD, did not detect Cel9A on the cell surface of the Δ*sprA* and Δ*sprT* mutants. Cel9A was accumulated in the periplasmic space of the Δ*sprA* and Δ*sprT* mutants. The result suggested the secretion defects of cell surface endoglucanases and the accumulation of them in the periplasmic space of the Δ*sprA* and Δ*sprT* mutants, which may be the reason for the decreased cell surface cellulase activities and the increased intracellular cellulase activities of the Δ*sprA* and Δ*sprT* mutants. The accumulation of T9SS substrates in the periplasmic space of the Δ*sprA* and Δ*sprT* mutants induced the over expression of DegQ, which is a member of the highly conserved HtrA family. Additionally, Wang et al. (2020) reported that “DegQ acted as a chaperone-protease facilitating refolding or degradation of periplasmic misfolded proteins in *C*. *hutchinsonii*.” Another unique feature of *C*. *hutchinsonii* is rapid cell motility over surfaces without flagella or type IV pili. It is speculated that gliding along the cellulose fiber facilitates efficient cellulose utilization (Stanier, 1942; Zhu and McBride, 2017). Deletion of *sprA* or *sprT* resulted in defects in colony spreading and individual cell motility. Gliding motility has been extensively studied in *F. johnsoniae*, in which SprB and RemA are propelled along the long axis of the cell on the cell surface by an unknown motor, generating cell motility. The secretion of SprB and RemA depends on the T9SS. The motility mechanism of *C*. *hutchinsonii* may be similar to that of the *F. johnsoniae*. The motility defects of the Δ*sprA* and Δ*sprT* mutants are likely resulted from the secretion defects of SprB-like adhesins to the cell surface. SprA was reported to be the outer membrane translocon of the T9SS (Lauber et al., 2018). However, the exact function of SprT in the T9SS remains unclear.

In conclusion, our study confirmed that CHU_0029 (SprA) and CHU_2709 (SprT) are both essential components of *C. hutchinsonii* T9SS. SprA and SprT are required for protein secretion, Ca^2+^ acquisition, cellulose degradation, and cell motility. Further studies focusing on the missing cell surface T9SS substrates of the Δ*sprA* and Δ*sprT* mutants would help to uncover the mysteries of crystalline cellulose degradation and Ca^2+^ assimilation mechanisms in *C. hutchinsonii*.

## Data Availability Statement

The original contributions presented in the study are included in the article/Supplementary Material, further inquiries can be directed to the corresponding author.

## Author Contributions

LG conceived and designed the experiments. LG and YT performed the experiments. LG, WZ, QQ, and XL analyzed the data. LG wrote the manuscript. LG and XL revised the manuscript. All authors read and approved the final manuscript.

## Conflict of Interest

The authors declare that the research was conducted in the absence of any commercial or financial relationships that could be construed as a potential conflict of interest.
