# Supplementary material for: Cytophaga hutchinsonii SprA and SprT Are Essential Components of the Type IX Secretion System Required for Ca2+ Acquisition, Cellulose Degradation, and Cell Motility
Source: Front Microbiol. 2021 Feb 12;12:628555. doi: 10.3389/fmicb.2021.628555 (PMC7906972; doi:10.3389/fmicb.2021.628555)
Supplement: Supplementary file 5 [file Data_Sheet_2.docx]

Supplementary Material

# Supplementary Data

**Table S1**

Table S1 Proteins with predicted immunoglobulin domain in *C*. *hutchinsonii* *^a^*

| Locus | Description | TIGRFAM family |
| --- | --- | --- |
| CHU_0938*^b^* | CHU large protein; uncharacterized | TIGR04183 |
| CHU_0939*^b^* | CHU large protein; uncharacterized | TIGR04183 |
| CHU_1157 | CHU large protein; candidate rhamnogalacturonan lyase | TIGR04183 |
| CHU_1162 | CHU large protein; candidate pectate lyase | TIGR04183 |
| CHU_1221*^b^* | CHU large protein | TIGR04183 |
| CHU_1634 | CHU large protein; uncharacterized | TIGR04131 |
| CHU_2040 | CHU large protein; esterase-related protein, CBM9 module | TIGR04183 |
| CHU_2041 | CHU large protein; candidate polyfunctional acetylxylan esterase/β-xylosidase/α-L-arabinofuranosidase, CBM9 module | TIGR04183 |
| CHU_2042 | CHU large protein, candidate xylanase, CBM9 module | TIGR04183 |
| CHU_2044 | CHU large protein; β-xylosidase/α-L-arabinofuranosidase related, CBM6 and CBM9 modules | TIGR04183 |
| CHU_2922*^b^* | CHU large protein; uncharacterized | TIGR04183 |

*^a^* Proteins containing immunoglobulin domain were identified by protein blast using National Center for Biotechnology information.

*^b^* Proteins were absent or decreased in abundance on the cell surface of the Δ*sprA* mutant and Δ*sprT* mutant.

# Supplementary Figures

**Fig. S1**


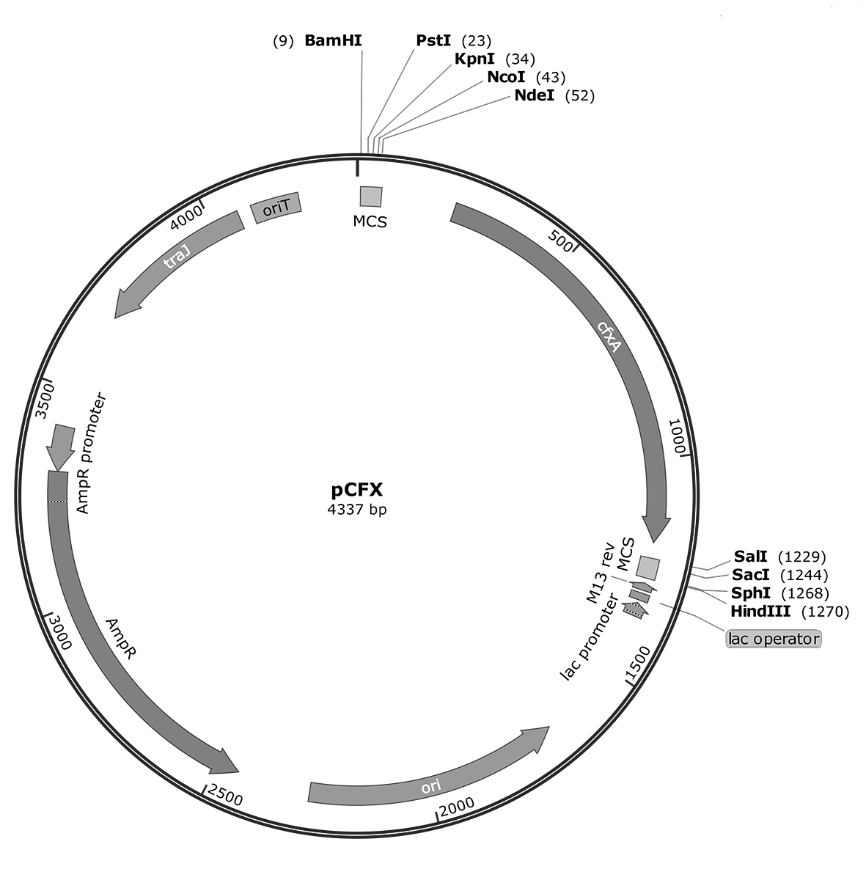


**Fig. S1** Map of the plasmid pCFX

**Fig. S2**


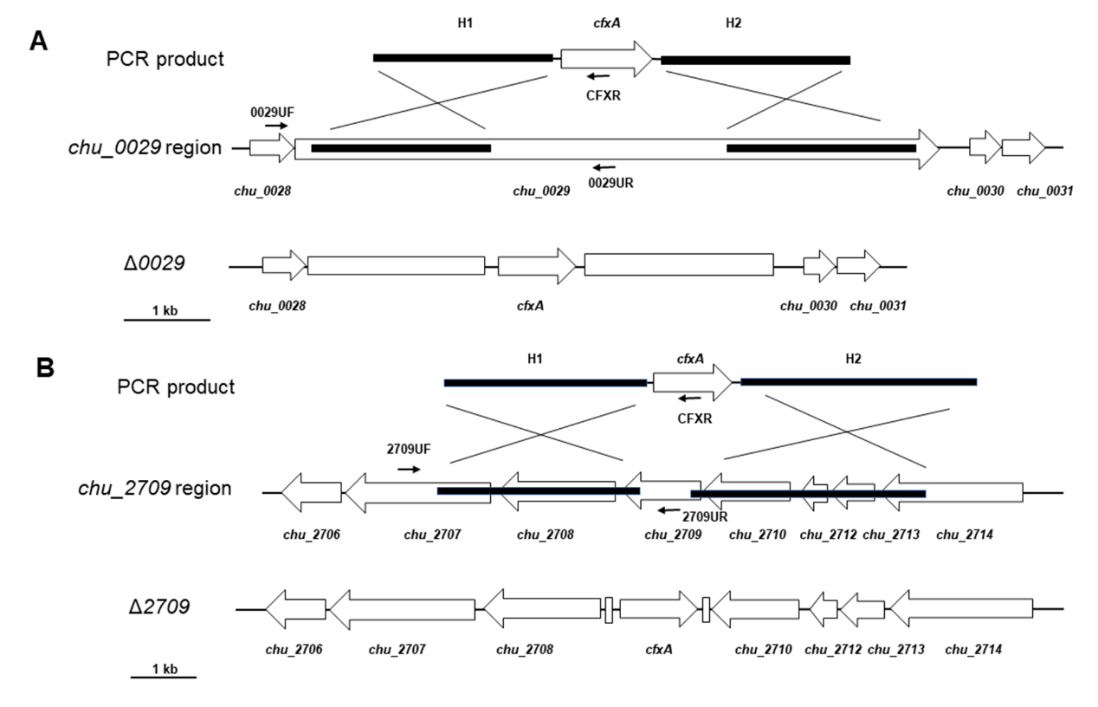


**Fig. S2** Illustration of the deletion processes of *chu_0029* and *chu_2709*. (A) The deletion of *chu_0029*. (B) The deletion of *chu_2709*. Black arrows show approximate locations and orientations of primers; black filled boxes indicate homologous arms; open arrowheads show arrangements and orientations of genes; open boxes indicate residual genes.

**Fig. S3**


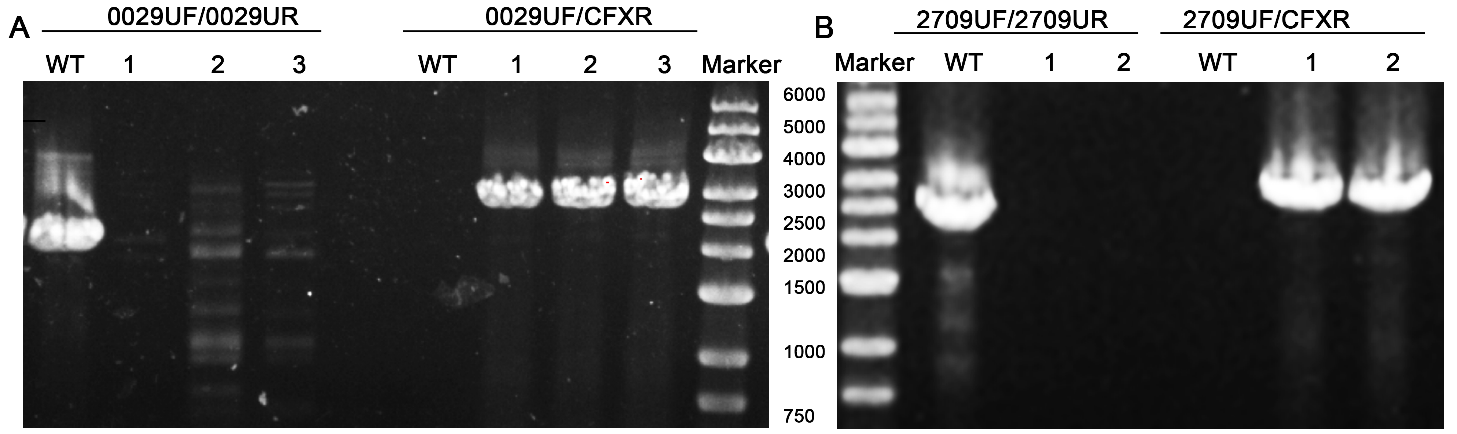


**Fig. S3** PCR verification the deletion of *chu_0029* (*sprA*) and *chu_2709* (*sprT*). (A) PCR verification the deletion of *sprA*. WT, wild type; 1-3, three transformants of the Δ*sprA* mutant. (B) PCR verification the deletion of *sprT*. WT, wild type; 1-2, two transformants of the Δ*sprT* mutant.

**Fig. S4**


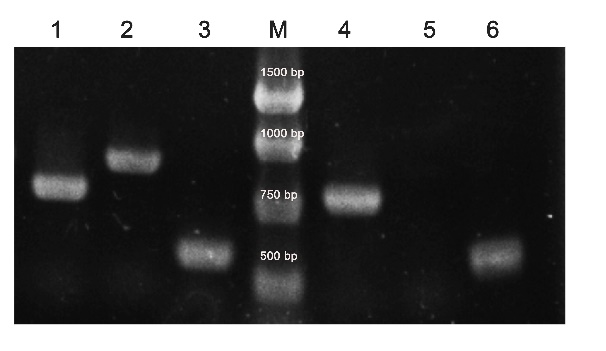


**Fig. S4** RT-PCR analysis the transcription of the surrounding genes of *sprA* (*chu_0029*) in the wild type and Δ*sprA* mutant. Lane 1, 2, 3 were performed with cDNA of the wild type, and Lane 4, 5, 6 were performed with cDNA of the Δ*sprA* mutant. Lane 1, 4 with primer pair of 0028F and 0028R, Lane 2, 5 with primer pair of 0029F and 0029R, Lane 3, 6 with primer 0030F and 0030R.

**Fig. S5**


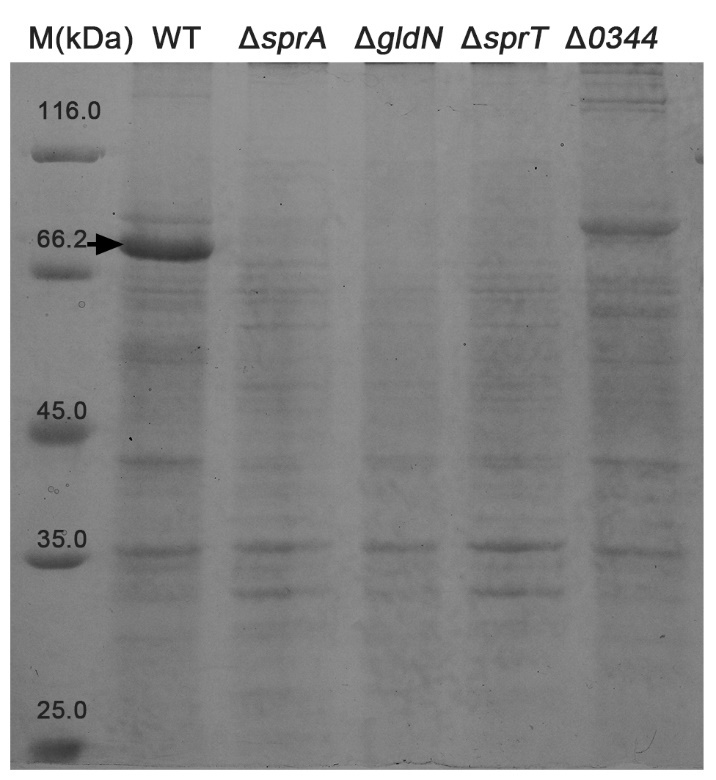


**Fig. S5** Full size of the SDS-PAGE gel of Figure 5A. The arrow indicated CHU_0344 identified by mass spectrometry.

**Fig. S6**


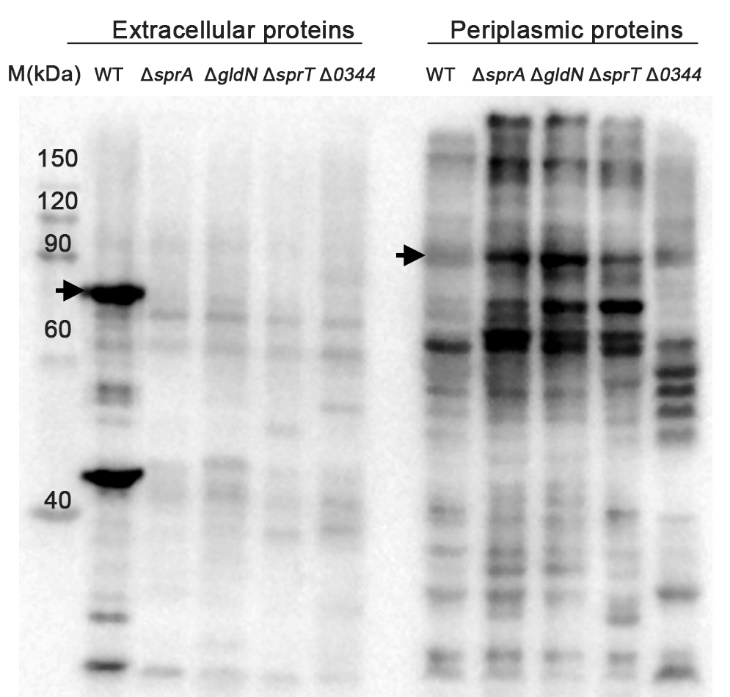


**Fig. S6** Full size of the Western blot figure of Figure 5B and 5C. The arrows indicated specific bands for CHU_0344 based on the molecular weight of the mature and primary product of CHU_0344.

**Fig. S7**


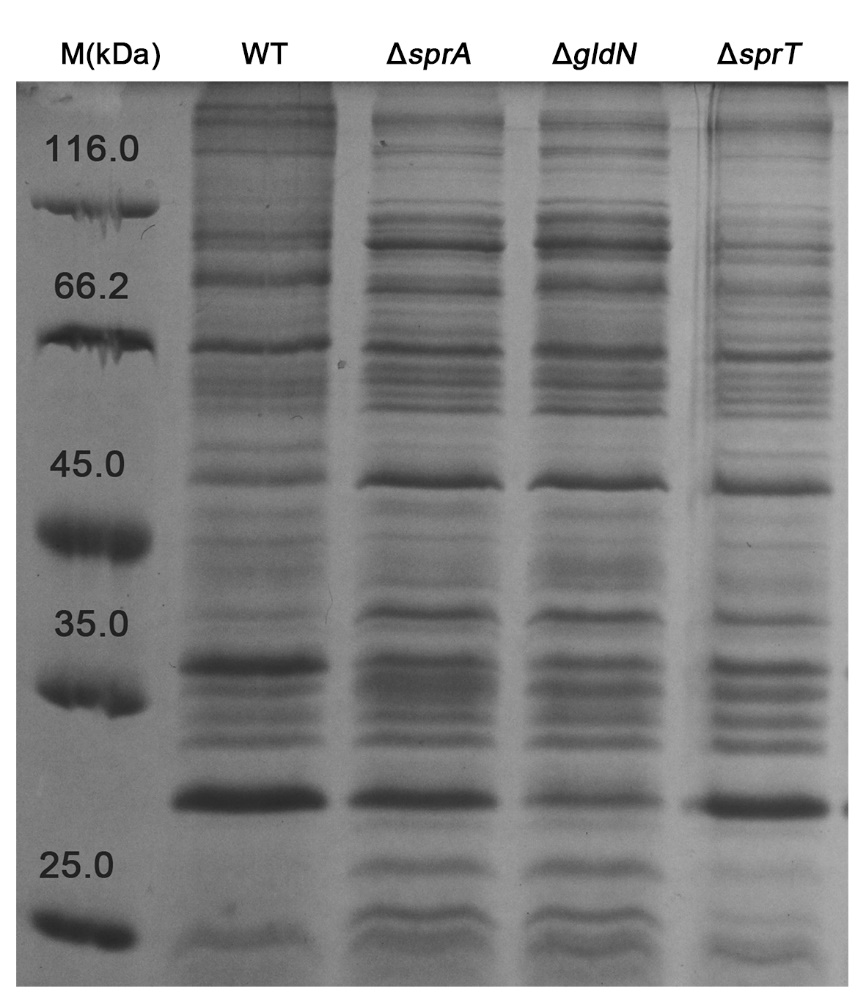


**Fig. S7** Full size of the SDS-PAGE gel of Figure 6.

**Fig. S8**


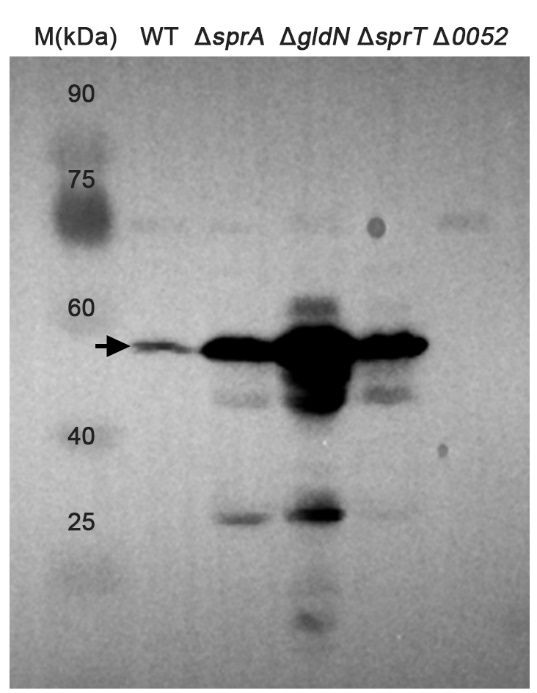


**Fig. S8** Full size of Western blot figure of Figure 7D. The arrow indicated the specific band for CHU_0052 based on the molecular weight of it.

**Fig. S9**


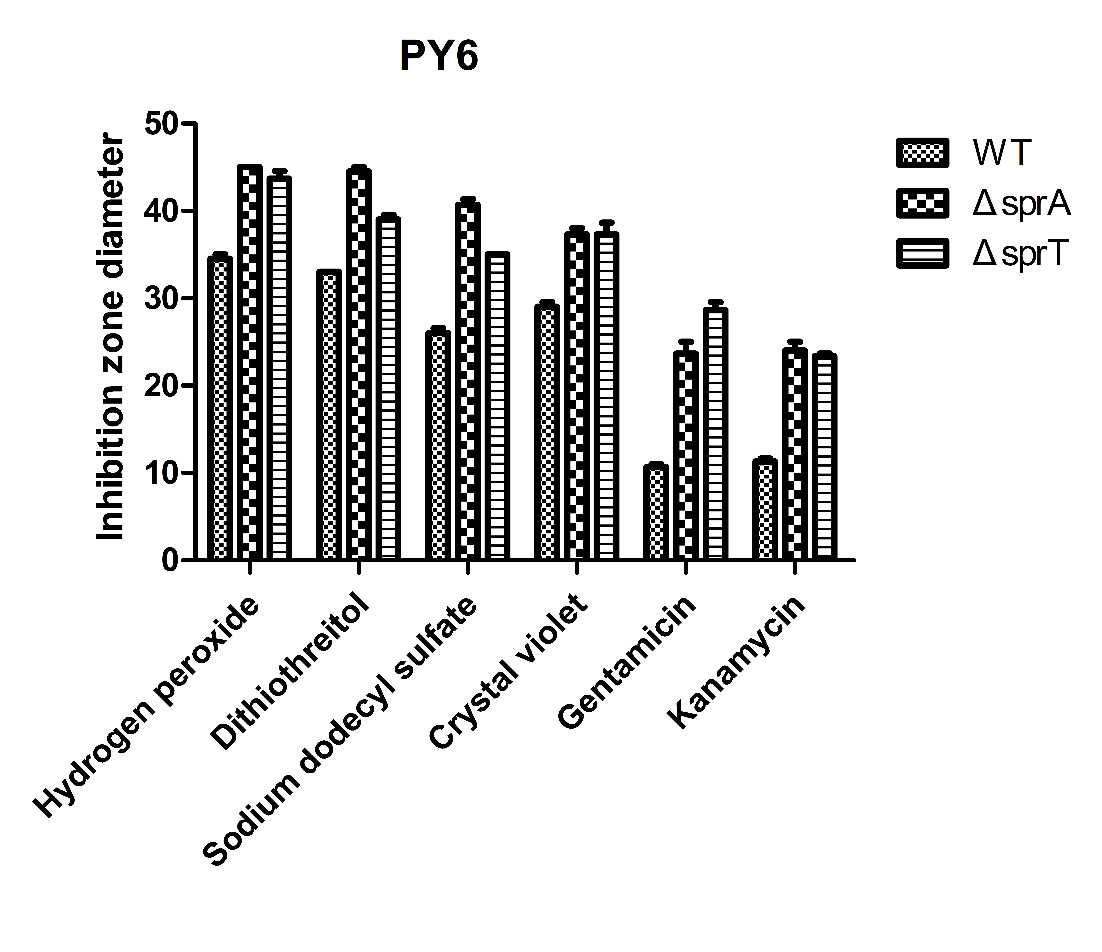


**Fig. S9** Inhibition zone diameters of the wild type, Δ*sprA* and Δ*sprT* mutants cultured in PY6 medium. The data shown are the mean values and SDs from three independent experiments.

**Fig. S10**


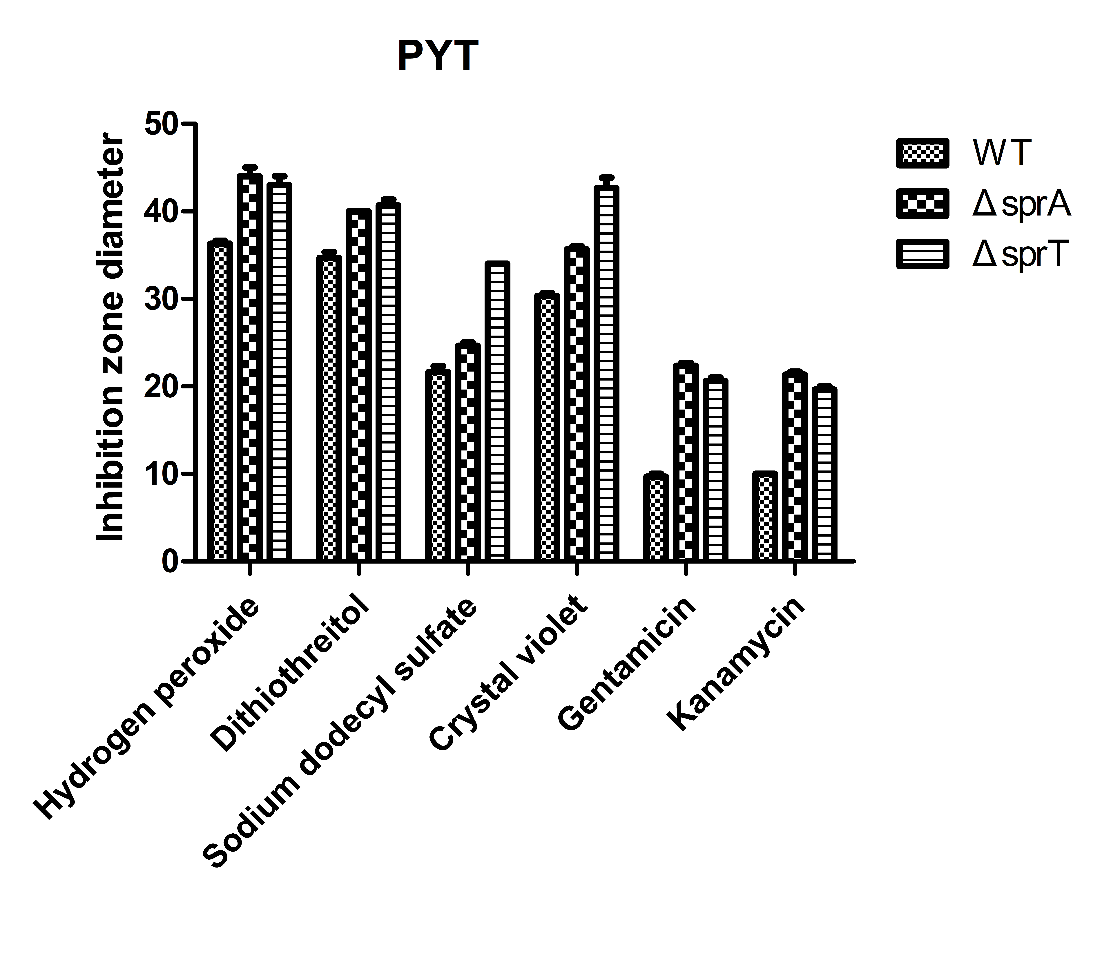


**Fig. S10** Inhibition zone diameters of the wild type, Δ*sprA* and Δ*sprT* mutants cultured in PYT medium. The data shown are the mean values and SDs from three independent experiments.

**Fig. S11**


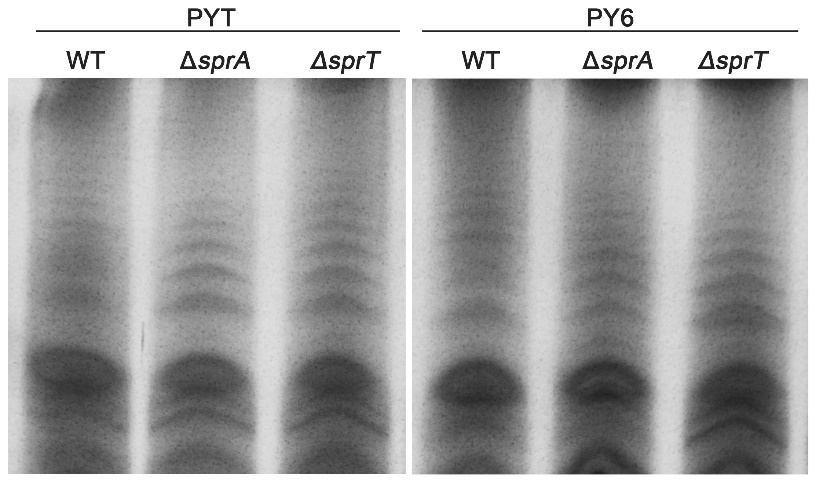


**Fig. S11** Lipopolysaccharide (LPS) structures of the wild type, Δ*sprA* and Δ*sprT* mutants. Strains were cultured in PYT medium or PY6 medium to mid-log phase.
